# Association of Game-Specific Performance of Young Skilled Basketball Players with Sensorimotor Factors of Agility Skills

**DOI:** 10.5114/jhk/202260

**Published:** 2025-04-30

**Authors:** Erika Zemková, Henrieta Horníková, Filip Skala, Gustáv Argaj

**Affiliations:** 1 Department of Biological and Medical Sciences, Faculty of Physical Education and Sports, Comenius University in Bratislava, Bratislava, Slovakia.; 2 Department of Track and Field and Sport Conditioning, Faculty of Physical Education and Sports, Comenius University in Bratislava, Bratislava, Slovakia.; 3 Department of Sports Games, Faculty of Physical Education and Sports, Comenius University in Bratislava, Bratislava, Slovakia.

**Keywords:** agility skills, change of direction speed, cognitive functions, decision-making, indicators of game performance

## Abstract

Reactive agility is one of the most important skills in basketball. However, the question remains to what extent the sensory and motor components of agility contribute to specific performance in the game. This study investigated the relationship between indicators of game-specific performance and perceptual-cognitive and physical aspects of agility performance in youth skilled basketball players. A group of 16 basketball players (age 15.5 ± 0.9 years) performed visual perception tasks, divided attention tasks, simple and choice reaction tests, along with Y-shaped and Lane agility tests. Their specific game performance was evaluated using the game statistics analysis. The visual perception score significantly correlated with assists (r = 0.850, p = 0.000), game efficiency (r = 0.760, p = 0.001) and total points scored (r = 0.715, p = 0.003). Coefficients of determination showed that visual perception explained 72.3% of the variance in assists, 57.8% in game efficiency and 51.1% in total points scored. These findings indicate that specific game performance in basketball is not associated with sensory and physical aspects of agility skills in youth players. An exception is visual perception, which plays a significant role in their performance. Players with better visual perception are able to dish out more assists, are more efficient in the game and score more points overall.

## Introduction

Basketball performance is determined by sensorimotor factors, including speed of responses to visual stimuli, running speed with changes of direction and explosive strength of the lower limbs (Horníková et al., 2023). More specifically, it is related to faster lane agility time, a longer standing long jump, weaker left side grip strength, more mobile hips, and a stiffer trunk (McGill et al., 2012). Core stability is further associated with assists, steals and agility, and subsequently agility is significantly correlated with steals (McGill et al., 2012). Of the combined National Basketball Association tests, only faster track agility time is significantly associated with basketball-specific performance, including minutes played, points, assists, and steals (McGill et al., 2012).

From this finding it is clear that agility is one of the most important skills in basketball. It addresses perceptual and decision-making factors on the one hand, and change of direction speed on the other (Young et al., 2002). The first factors include the ability of players to predict an in-game event that affects their in-game movement, process visual information in competitive games, reorganize the playing patterns of the opposing team or teammates, and knowledge of the likely movements of other players based on previous experience with the game, while the second factors are leg muscle properties, linear sprint speed and technique (Young et al., 2002). Later, this model was divided into three components such as cognitive, physical, and technical (Young et al., 2015). The first component involves anticipation, visual scanning, pattern recognition, and situational awareness, the second involves reactive power and strength, linear sprint speed and core strength, and the third involves step adjustment for acceleration and deceleration, foot placement, posture, and body lean (Young et al., 2015).

In contrast to the well-investigated physical aspects of agility skills, less is known about their sensory component. Perceptual-cognitive abilities refer to the player's ability to effectively devote attentional resources in response to movement patterns of crucial situations in a fast dynamic environment (Faubert and Sidebottom, 2012). They are better in elite athletes than in novices or non-athletes, especially in combat and team sports. This can be observed in better choice reaction time to monocular stimuli (Paulus et al., 2014), namely visual reaction time, decision-making, spatial orientation, focused attention, perceptual speed, prediction and estimation of the direction and speed of a moving object (Kioumourtzoglou et al., 1998), as well as faster responses to stimuli displayed in both peripheral and central locations (Ando et al., 2001). This can be attributed to their better visuomotor skills (Gao et al., 2015), visual search behavior with fewer fixations of longer duration and prolonged quiet eye periods (Mann et al., 2007), accommodative ability and saccadic eye movements (Jafarzadehpur et al., 2007), capture of perceptual cues as reflected in response accuracy and response time (Mann et al., 2007), visual functions including contrast sensitivity, contour and random dot stereoacuity, and monocular visual acuity (Laby et al., 2011), static stereo (Boden et al., 2009) and dynamic visual acuity during free movement of the eyes due to their excellent ability to follow moving targets with the eyes (Quevedo-Junyent et al., 2011; Uchida et al., 2012), and the ability to learn to process complex dynamic visual scenes (Faubert, 2013).

In addition to sports specialization and the level of expertise, the sensory aspect of athletes' performance also depends on their age. Excellent perceptual and cognitive abilities are already observed in 9-year-old soccer players (Ward and Williams, 2003). Spatial perception in young athletes is related to their alerting efficiency. As shown in a group of 16-year-old badminton, table tennis, and tennis players, the faster and more accurate their spatial perception response, the higher their alerting efficiency (Wang et al., 2024). Skilled 11- to 13-year-old basketball players show excellent stereoscopic vision and distance visual acuity, good visual reaction times and horizontal visual fields, with many of them having eye-hand cross-dominance (Sillero Quintana et al., 2007). This ability continues to improve with age. For instance, response time to stimuli displayed in the peripheral visual field is shorter in expert and U19 than U15 basketball players (Chaliburda et al., 2023). Quiet eye time and total fixation duration are longer and the number of fixations is less in adult professional basketball players compared to players under 16 years of age (Rui et al., 2023). Similarly, visual tracking is superior in experienced U20 than U17 and U15 soccer players (Alves et al., 2015). With this in mind, perceptual-cognitive function plays an important role in the game performance of young players.

However, the question remains to what extent the sensory component of agility skills contributes to specific basketball performance in the game. Herein, we investigated the relationship between game-specific performance indicators and perceptual-cognitive and physical aspects of agility in youth skilled basketball players. We hypothesized that visual perception would positively correlate with game efficiency, total points scored, and assists.

## Methods

### Participants

Sixteen youth basketball players (age 15.5 ± 0.9 years, body height 1.86 ± 0.08 m, body mass 69.9 ± 9.7 kg) from a local academy participated in this investigation. Players were from the age groups of U15 and U17. They participated in organized basketball training for 5.4 ± 0.5 years. They trained 8.5 hours per week (five game training sessions lasting 90 min on the basketball court and two conditioning training sessions lasting 30 min in the gym) plus friendly or official matches on the weekend. There were no neuromuscular injuries or any disorders among the study participants. Written informed consent was obtained prior to the study.

### Design and Procedures

This study was designed as a cross-sectional experiment. It examined the association of game statistics with the physical and perceptual- cognitive aspects of agility performance of youth basketball players. Data collection was implemented in the competitive phase of the season. Players underwent one testing session on the same day. All assessments were undertaken by experienced examiners during a training session (between 4 and 7 p.m.) in the gymnasium, under the same conditions for all participants. A 10-min warm-up that included jogging, a series of dynamic stretching exercises, multiple acceleration runs, and potentiation exercises was performed prior to the testing sessions. Players were asked to arrive at the testing sessions well-rested and hydrated, without any intense training within the preceding 24 h. This research was in accordance with the ethical standards on human experimentation stated in compliance with the 1964 Helsinki Declaration and its subsequent modifications. This project was approved by the ethics committee of the Faculty of Physical Education and Sports, Comenius University in Bratislava, Bratislava, Slovakia (approval code: 2/2023; approval date: 07 February 2023).

### Measures

For game data collection, observation and expert assessment were used. Indicators of game performance were obtained from the Genius Sports and FIBA websites. The method of the evaluation of Game Efficiency (GE) determined the individual effectiveness of game skills in a match. This method was performed as follows:

1) evaluation of critical cases from the perspective of performance in the most significant game skills (Flanagan, 1954); 2) GE was determined as the difference between the index of positive critical cases (IPCC) and the index of negative critical cases (INCC); 3) the value of GE was determined in absolute values (the number of minutes played by individual players was not taken into account); 4) the index of positive critical cases was determined as the sum of the indices of individual positive critical cases; 5) the index of a positive critical case was determined as the product of its frequency of occurrence and the significance index; 6) the overall value of the index of positive critical cases was determined by the relationship: IPCC = (TNP * 1) + (OR * 0.7) + (OA * 0.5) + (F * 0.5) + (DR * 0.7) + (DA * 0.5) + (GP * 0.7) + (BS *0.5). An overview of positive critical cases, their designation, and significance index are shown in Table 1.

**Table 1:** Description of game statistics.

Game indicators	Abbreviation	Significance index
Total number of points	TNP	1
Offensive rebounds	OR	0.7
Offensive assists	OA	0.5
Fouls	F	0.5
Defensive rebounds	DR	0.7
Defensive assists	DA	0.5
Gained possessions	GP	0.7
Blocked shots	BS	0.5

### Simple and Choice Reaction Tests

The diagnostic system FiTRO Reaction Check (FiTRONiC, Bratislava, Slovakia) was used to measure simple and choice reaction time. This system consisted of two pressure switches connected to the interface and the computer. Players were asked to press a switch on the table as quickly as possible to a single visual stimulus (a simple reaction time test) or to one of two stimuli (a choice reaction time test). A stimulus consisting of a red circle placed on a white background was used for the simple reaction test. During the choice reaction test, players were required to select and react to one of two visual stimuli by pressing the switch of matching color. When the red circle appeared on the screen, the player was required to press the right switch. Conversely, the left switch was required to be pressed when the blue circle appeared. Both hands maintained continuous contact with the switches to minimize the involvement of the motor component of movement. Both tasks consisted of two trials with 20 responses. Reaction times to incorrect responses were not taken into account. The results of these tests were the trials with the lowest mean reaction times.

### Y-Shaped Agility Test

A Witty timing system (Microgate, Bolzano, Italy) was used to measure reactive agility. This system consisted of single-beamed timing gates and a LED sensor paired with the timing system. Gates were separated by 1.5 m in width, and the height of the beam was 1.2 m. The LED sensor was used as a visual trigger for the change of direction of participants. They started the test 50 cm behind the starting line and executed a 5-m linear sprint. Next, they executed the 45-degree change of direction to either the left or the right side and proceeded 5 m to the finish gates. The green arrow served as a stimulus to show the direction of the movement. This arrow occurred with a delay of 0.5 s from the break of the starting timing gate. In total, three repetitions were conducted, and the lowest time was selected for subsequent data analysis. This test was found to be reliable and valid for basketball players (Lockie et al., 2014; Oliver and Meyers, 2009).

### Lane Agility Test

This test was implemented to evaluate the change of direction speed ability of the players. The cones were placed at the four corners of the “key” on a standard-sized basketball court. Players started 50 cm behind the starting line (cone D), which was positioned at the left-hand corner of the free throw line facing the baseline. They were instructed to sprint forward to cone A, shuffle right to cone B, run backward to cone C at the free throw line, and shuffle left to cone D. Then, they changed directions to shuffle to the right back to cone C, sprint forward to cone B, shuffle left to cone A, and finish running backwards to the original starting position (cone D). The time of the test was measured using a single beam electronic system Witty Gate (Microgate, Bolzano, Italy). Three trials were allowed for each participant, and the lowest time was taken for the analysis. The lane agility test has been shown to be a reliable assessment of change of direction ability in adolescent basketball players (ICC = 0.88, CV = 7.3%) (Stojanović et al., 2019).

### Divided Attention Task

Players stood in front of the four LED sensors (Witty SEM, Microgate, Bolzano, Italy). The height of the sensors was adjusted to the chests of the players. Players stood at the length of their upper extremities in relation to the sensors. Two sensors showing the symbols were in front of the player, and two representing the “yes” and “no” responses were at the 30 degree angle by the player. The LED sensors repeatedly displayed two symbols at the same time. Participants had to decide whether the symbols were of the same shape. If this was true, they put their hand close to the left sensor, representing “yes”. Conversely, they put their hand close to the sensor “no” if symbols were of different shapes. The outcome of this test was a ratio between total time and the number of errors. The best of three trials was considered a result of the divided attention test.

### Visual-Perception Readiness Test

Participants were seated comfortably in the room with the testing sheet on the desk. The researcher explained the test verbally to each participant. The testing sheets included pictures of six squares, each containing six circles (2 yellow, 2 red, and 2 green). The goal of the task was to determine which two circles of identical color in each square were the widest apart in the shortest time possible. Participants were instructed to solve the test sheet with the pencil continuously from top to bottom. The visual-perception readiness test consisted of one familiarization trial including 15 responses and one testing trial including 70 responses. If they realized that a mistake was made, they could correct them in the remaining time. The total solution time of the test was 70 s. The outcome of the test was the number of correct responses.

### Statistical Analysis

The statistical program used for the analysis of data was IBM SPSS for Windows (version 23.0, Inc., Chicago, IL, USA). The Shapiro-Wilk test for homogeneity of variance revealed that the data were normally distributed. However, due to the ordinal character of some correlated data, the Spearman´s rank correlation coefficient (r) was used to determine the relationship between the variables of perceptual-cognitive and agility tests and indicators of game performance. The correlation was considered as no correlation (0.0 > 0.1), low (0.1 > 0.3), medium (0.3 > 0.5), high (0.5 > 0.7), or very high (0.7 > 1.0) (Kuckartz et al., 2013). The coefficient of variation (R^2^) computed as a square of the correlation coefficient was used to indicate the fraction of the total variance in the dependent variable explained by the independent variable. The level of significance was set at *p* < 0.05.

## Results

The simple reaction time was 294 ± 27 ms, choice reaction time was 412 ± 26 ms, reactive Y-shaped agility time was 2.1 ± 0.1 s, pre-planned lane agility time was 12.6 ± 0.8 s, the index in the divided attention task was 8.9 ± 5.4, and the visual perception score was 30.9 ± 5.0.

As for the game performance indicators of basketball players, the game efficiency received 9.9 ± 6.8 points, assists 24.7 ± 15.3 points, rebounds 46.2 ± 37.2 points, and the total number of points was 62.9 ± 69.9.

**Figure 1:**
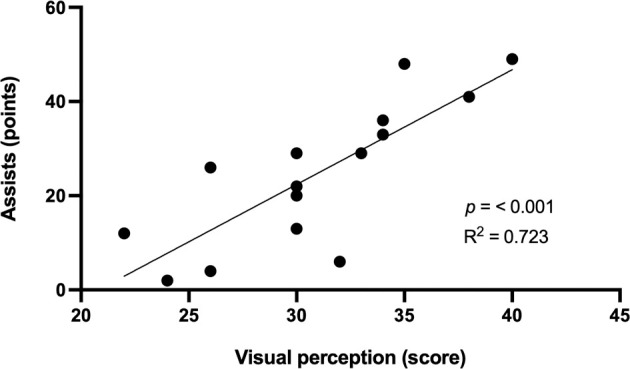
Relationship between the visual perception score and the number of assists.

**Figure 2:**
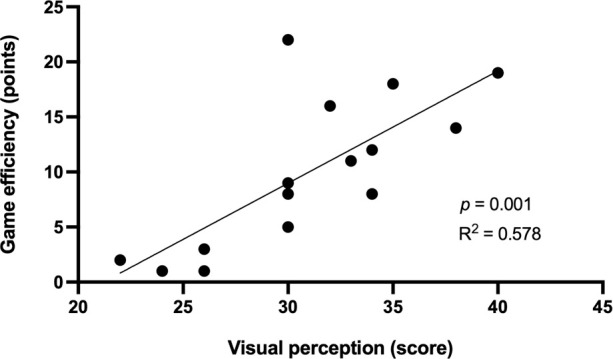
Relationship between the visual perception score and game efficiency.

**Figure 3:**
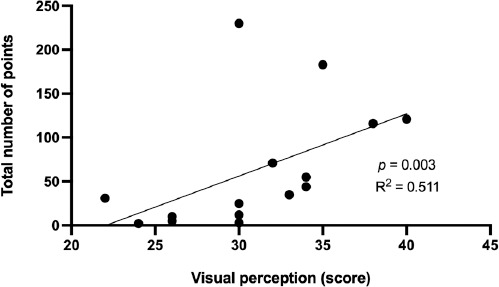
Relationship between the visual perception score and total points scored.

The visual perception score was significantly correlated with assists (r = 0.850, *p* <0.001), game efficiency (r = 0.760, *p* = 0.001), and total points scored (r = 0.715, *p* = 0.003) (Table 2). The coefficients of determination showed that visual perception explained 72.3% of the variance in assists, 57.8% in game efficiency and 51.1% in total points scored (Figures 1–3).

**Table 2:** Correlations between indicators of game performance and variables of perceptual-cognitive and motor abilities.

	Game efficiency		Total number of points		Assists		Rebounds	
	*r*	*p*	*r*	*p*	*r*	*p*	*r*	*p*
Simple reaction time	0.010	0.972	−0.109	0.698	−0.104	0.619	−0.102	0.699
Choice reaction time	0.156	0.579	0.093	0.742	−0.148	0.599	0.268	0.335
Divided attention index	−0.116	0.680	−0.257	0.355	−0.256	0.358	−0.100	0.723
Visual perception score	0.760	0.001	0.715	0.003	0.850	<0.001	0.314	0.254
Y-shaped agility time	−0.183	0.515	−0.159	0.571	−0.386	0.155	−0.275	0.322
Lane agility time	−0.299	0.279	−0.250	0.368	−0.063	0.824	−0.285	0.304

## Discussion

The most important finding is the significant relationship between the visual perception score and specific performance indicators such as assists, game efficiency and total points scored. This indicates that the perceptual functions that were measured under time pressure reflected the perception of players in a sport-specific environment. It points to their ability not only to react faster, but also more effectively under game-specific conditions. Assisting players with passes actually requires scanning the environment and quick decision-making processes regarding the perception of the players' position and space. More space for defensive players increases the likelihood that offensive players will pass the ball into a promising scoring opportunity. This is also partially true of the scoring process. Players must be aware of the surrounding space and decide to shoot in a very short time. Therefore, visual perception and fast decision-making based on this domain may explain the higher game efficiency of players with higher scores on the visual perception test.

In this context, Mangine et al. (2014) identified the relationship between visual tracking speed and basketball-specific performance represented by assists, steals, and assists/turnovers, and possibly also turnovers. This indicates that visual tracking speed is related to a player’s ability to see and respond to various stimuli on the basketball court. This can result in better plays as presented by a greater number of assists and steals and a lower number of turnovers. Players are able to follow movements of teammates and opponents on the field and this allows them more time to respond to the demands of a given situation. However, it should be taken into account that these were professional basketball players on a National Basketball Association aged from 19.4 to 30.7 years (Mangine et al., 2014) compared to 15.5-year-old skilled basketball players in our study. Their better visual perception scores were related to more assists and higher game efficiency. It is very likely that as their perceptual-cognitive functions improve with increasing age and experience, their game performance will also improve. Experienced basketball players use a simple and efficient visual search strategy, including more fixations and longer fixation duration in more informative areas than novices (Jin et al., 2023). They have a shorter fixation trajectory and are mainly focused on the area of key information (Jin et al., 2023). Experts are able to monitor the prediction performance by obtaining important shooting information such as the player's body (Li et al., 2023). However, they were 20-year-old athletes with nine years of playing experience (Jin et al., 2023; Li et al., 2023) compared to five years of basketball training of players in our study. Similarly, 20-year-old basketball experts are better at prediction and selective attention of both relevant and irrelevant information cues (Kioumourtzoglou et al., 1998). Skilled 25-year-old basketball players with 13 years of playing experience have faster response times and higher response accuracy when watching video clips of basketball scenarios with full-screen control, a moving mask (peripheral vision only), and a moving window (central vision only) (Ryu et al., 2013). Their gaze behavior is less affected by gaze-contingent manipulations, indicating that they use the remaining information to maintain their normal gaze behavior (Ryu et al., 2013). Twenty-three-year-old basketball players playing at competitive levels over 10 years exhibit a better near point of convergence, halo discriminability, positive fusional vergences, and eye-hand coordination, most likely due to the systematic engagement of those skills during training (Vera et al., 2020). From this it is obvious that these specific skills are better in older and more experienced players compared to their younger counterparts. Specifically, peripheral perception is better in expert and U19 than U15 basketball players in terms of the field of vision, visual left and right angles, and left and right eye reaction time (Chaliburda et al., 2023). Therefore, even in our group of 15.5-year-old basketball players, it is possible to assume an improvement in their perceptual-cognitive functions with training experience, which should subsequently be reflected in their better game performance.

Furthermore, a moderate but non-significant correlation was shown between visual perception scores and the number of rebounds. It could be suggested that rebounds in basketball depend more on the height and physical abilities of players than their perceptual-cognitive and visual skills. Moreover, a moderate relationship was also found between the number of assists and time in the reactive Y-shaped agility test. This may indicate lower specificity of the reactive task, which requires a response to light stimuli in a 45-degree direction. This suggestion is in accordance with the study by Barrera-Dominguez et al. (2024) who demonstrated that linear and change of direction speed in basketball players was determined by a long stretch-shortening cycle (SSC) in a vertical direction in females, whereas in males it was a short SSC predominantly in a horizontal direction (e.g., a triple hop test), except for the change of direction at the angle of 45º. Basketball requires visual perception of the opponent and the ball, with changes of direction in the defensive phase often greater than 180 degrees. It also follows that sensory functions play an essential role in reactive agility and, consequently, in game-specific performance of youth basketball players. These findings are consistent with previous ones that demonstrated significant relationships among various reactive agility tasks and acceleration and sprint speed, cognitive ability of divided attention, reactive and explosive strength of the lower limbs, and speed of responses to slowly generated visual stimuli in basketball players (Horníková and Zemková, 2022; Horníková et al., 2023).

However, there were no significant relationships between measures of game performance and time in the pre-planned lane agility test or other sensorimotor variables, including simple and choice reaction time. Similarly, Mangine et al. (2014) reported no relationship between simple and choice reaction time and any of the basketball-specific performance measures. Measuring simple and choice reaction time to visual stimuli displayed on a computer monitor may not reflect stimuli under the conditions of a basketball game where players respond to the movement of teammates and opponents, or the ball. The mean simple and two-choice reaction time in the group of youth basketball players was 294 ms and 412 ms, respectively. These results are comparable to simple and two-choice reaction time (298 ms and 426 ms, respectively) of 20-year-old competitive men's basketball players (Horníková and Zemková, 2022; Horníková et al., 2023). Better simple reaction time (SRT) was achieved by skilled 13-year-old basketball players (240 ms) using the Visual Lab programme (Cedrus Corporation, San Pedro, CA) that was run on a laptop with a 15-in. TFT screen (Sillero Quintana et al., 2007). These variations can be explained by differences in SRT latencies, in part due to timing delays introduced by the computer hardware and software used for SRT measurement in different laboratories (Woods et al., 2015). This can increase measured simple reaction time latencies by up to 100 ms (Neath et al., 2011).

Taking into account interrelationships between the visual system and the sensory-motor coordination of the whole body (Jafarzadehpur et al., 2007), more attention should be paid primarily to the development of the perceptual-cognitive component of agility skills in youth athletes (Zemková and Hamar, 2018). Both the speed of decision-making and change-of-direction speed contribute to athletes' agility, albeit to varying extent depending on sport specialization, their performance level, testing conditions, and so forth (Zemková, 2017). Estimating the contribution of sensory and motor components to agility under conditions close to the demands of a given sport is useful for distinguishing between and within group differences, assessing acute changes after exercise as well as long-term adaptation after sport-specific training. For instance, combined agility and balance training improves the motor rather than the sensory aspect of agility in basketball players (Zemková and Hamar, 2010). This was confirmed by a significant correlation between an increase in movement speed and the reduction in agility time after training. This indicates that training in competitive athletes is not sufficient to improve the perception and decision-making component of agility. Although training programs are designed to improve sensory function, they usually consist of exercises that also include motor tasks. As a result, improvements in agility performance are often attributed to improvements in sensory function; however, it can equally be attributed to the improvement of motor functions (Zemková, 2022). Therefore, it is important to understand the relationship between perceptual-cognitive and physical aspects of agility with game-specific performance indicators, especially in youth basketball players. This can be useful not only for a more specific assessment of their agility performance, but also for designing training programs targeting key game-specific skills.

A certain limitation of this study is the diagnostic system used to measure simple reaction time, as its values were higher compared to those in the above-mentioned studies. The reason could be the poor calibration of computer hardware, and the weak precise software used in these measurements. Furthermore, the perceptual-cognitive functions of players were assessed using tests requiring mainly responses to non-specific stimuli. Testing methods with higher ecological validity would likely more accurately reflect players' game performance. Cognitive functions of interest such as attention, perception, memory, executive function, processing speed, or spatial processing could not be tested in isolation, but rather in the context of more complex tasks. Thus, the question remains to what extent these domains would contribute to game performance of youth basketball players. In addition to answering this question, further research should use an optical tracking system that could provide a deeper analysis of the players' game performance.

## Conclusions

Visual perception, as one of the components of agility, plays a significant role in game performance of basketball players. This can be corroborated by significant correlations between the visual perception score and assists, game efficiency and total points scored. Visual perception explained 72.3% of the variance in assists, 57.8% in game efficiency and 51.1% in total points scored. This means that better visual perception of players was reflected by more positive play on the basketball court. Therefore, basketball training should focus on the development of visual perception, which is largely involved in the game-specific performance of youth basketball players.
